# Exosomal miR‐106a‐5p derived from intermittently hypoxic non‐small‐cell lung cancer increases tumor malignancy

**DOI:** 10.14814/phy2.16157

**Published:** 2024-07-31

**Authors:** Jie Ren, Zhuan Jin, Yongjie Huang

**Affiliations:** ^1^ Department of Respiratory Medicine The First Affiliated Hospital of Zhengzhou University Zhengzhou Henan China

**Keywords:** Exosomal miR‐106a‐5p, non‐small cell lung cancer, obstructive sleep apnea, PTEN, STAT3

## Abstract

Intermittent hypoxia (IH) is a hallmark of obstructive sleep apnea (OSA), which is related to tumorigenesis and progression. We explored the possible mechanisms by which OSA may promote the development of non‐small cell lung cancer (NSCLC). In this study, NSCLC cells with and without miR‐106a‐5p inhibition were exposed to IH or room air (RA), and subsequently, exosomes were extracted and identified. Macrophages were incubated with these exosomes to detect the expression of the STAT3 signaling pathway and M2‐type macrophage markers, as well as the effect of the macrophages on the malignancy of NSCLC cells. A nude mouse tumorigenesis model was constructed to detect the effects of exosomal miR‐106a‐5p on M2 macrophage polarization and NSCLC cell malignancy. Our results showed that IH exosomes promoted the polarization of M2 macrophages, thereby promoting the proliferation, invasion, and metastasis of NSCLC cells. Further, Based on microarray analysis of RA and IH exosomes, we discovered that miR‐106a‐5p, transferred to the macrophages through exosomes, participated in this mechanism by promoting M2 macrophage polarization via down‐regulating PTEN and activating the STAT3 signaling pathway in vitro and in vivo. For patients with NSCLC and OSA, exosomal miR‐106a‐5p levels showed a positive relation to AHI. Exosomal miR‐106a‐5p represents a potential therapeutic target among patients with concomitant cancer and NSCLC.

## 1 INTRODUCTION

Obstructive sleep apnea (OSA) is one of the most common sleep apnea disorders, currently affecting 2%–4% of adults, and its incidence is increasing year by year worldwide (Young et al., 1993). OSA is characterized by the recurrent complete and/or incomplete obstruction of the upper airway during sleep, leading to intermittent hypoxia (IH) and sleep fragmentation. In recent years, the relationship between OSA and tumor development has attracted substantial attention. In 2012, a follow‐up study of 1522 subjects in Wisconsin, USA, found that tumor mortality was positively correlated with the severity of OSA. The tumor mortality of patients with mild, moderate, and severe OSA was 1.1, 2.0 and 4.8 times that of the control group, respectively (Miller et al., 2013). Another study showed that after a 20‐year follow‐up period, there was a significant increase in tumor mortality (HR, 3.4; 95% CI:1.11–10.2) and incidence (HR, 2.5; 95% CI:1.2–5.0) in patients with moderate to severe OSA (Marshall et al., 2014). According to a multicenter cohort study, a 10% increase in the nighttime period of oxygen saturation below 90% was associated with a 1.07‐fold increase in tumor incidence (Campos‐Rodriguez et al., 2013). Almendros et al. showed that the size and weight of the tumors in the IH group were 2.5 times that of the room‐air (RA) group (Almendros et al., 2016). Compared with the RA group, IH appeared to promote the metastasis of melanoma into the lungs, as the number and weight of these lung metastases were significantly increased in the IH group (Li et al., 2018). Almendros et al. (2014) showed that IH promoted the proliferation, migration and invasion abilities of TC1 cells by a mechanism that is postulated to be associated with tumor‐related macrophages.

The immune system and its cells could play an important role in the possible mechanisms behind OSA and tumor development. Macrophages are usually divided into two polarization phenotypes, the classical M1 type and the alternative M2 type (Brown et al., 2017). The M1 macrophage is involved in the inflammatory responses, pathogen clearance, and antitumor immunity (Chanmee et al., 2014). In contrast, the M2‐type macrophages play an important role in the remodeling of the extracellular matrix by driving neovascularization, mediating apoptosis, and inhibiting adaptive immune responses. These functions, in turn, can ensure the survival, proliferation, invasion, and metastasis of tumor cells (Brown et al., 2017). M2‐type macrophages also promote tumor cell growth by expressing high levels of anti‐inflammatory cytokines, such as IL‐10, as well as increasing Arginase 1 activity (Wang, Cao, et al., 2015). And, other studies found that the STAT3 signaling pathway could promote the polarization of M2 macrophages, and its downstream targets are IL‐6, CXCR4 and CCL2 (Ham et al., 2018).

Exosomes are extracellular vesicles that have a lipid bilayer structure, with a diameter of 40–150 nm, that are secreted by cells (Thery et al., 2002). Exosomes carry specific proteins, lipids, functional messenger RNAs (mRNAs), small molecule RNAs (miRNAs) and long non‐coding RNAs (lncRNAs) as well as other bioactive substances, which play an important role in cell communication in vivo (Raposo & Stoorvogel, 2013). Recent studies have suggested that hypoxia may promote tumor progression by altering the secretion of exosomes thereby regulating cellular communication (King et al., 2012). This allows exosomes from tumor cells to contribute to tumor development by communicating between the tumor and the surrounding stromal tissues, activating proliferation and angiogenesis pathways, initiating pre‐metastatic sites, and promoting immunosuppression (Mincheva‐Nilsson & Baranov, 2014).

MiRNAs are a class of small non‐coding RNAs 19 to 25 nucleotides long (Schickel et al., 2008). They control the fate of cells by acting on the mRNAs of proteins involved in cell signaling pathways (Avery‐Kiejda et al., 2014). Numerous studies have shown that exosomes promote cell migration (Zhang et al., 2010), angiogenesis (Mao et al., 2015) and tumor metastasis (Yamada et al., 2014) by transporting miRNAs, leading to the malignant transformation of normal cells in vivo and in vitro. However, the role of exosomal miRNAs in the promotion of tumor development by IH has not been studied. Similarly, the downstream signaling pathway of exosomal miRNAs and the possible involvement of macrophage polarization has not been investigated.

## 2 METHODS

### 2.1 Cell culture

Human non‐small cell lung cancer (NSCLC) cells (lung adenocarcinoma A549 cells and lung squamous NCI‐H226 cells) were obtained from Aspen Biotechnology (Wuhan, China). They were maintained in 4 mL RPMI‐1640 medium (Hyclone‐SH3080, USA) containing 10% fetal bovine serum (BI‐04–001‐1ACS, Israel). Human THP1 cell line was differentiated into macrophages by a 24 h incubation in the presence of 100 ng/mL PMA (Sigma, P1585).

### 2.2 Cell IH protocol and exosome isolation

Human lung adenocarcinoma A549 cells were cultured in 10% FBS/RPMI‐1640 medium (Hyclone‐SH3080, USA). When the confluency reached 80%, the cells were collected and treated to IH (30 min 5% O_2_ followed by 30 min 21% O_2_ balanced in 5% CO_2_, 1 cycle/h) or RA (21% O_2_ balanced in 5% CO_2_) for 48 h (Almendros et al., 2014). 30 mL of the cell culture was collected and centrifuged at 300 × *g* for 10 min, 2000 × *g* for 10 min, and 10,000 × *g* for 30 min to remove residual cells and debris. The exosomes were then resuspended and collected by centrifugation at 100,000 × *g* for 70 min (Wang, Yao, et al., 2015).

### 2.3 Dual luciferase reporter assay

The 3′‐ untranslated region (UTR) region of PTEN was inserted behind the luciferase reporter gene in the pGL3‐basic vector to construct the luciferase plasmids. The miR‐106a‐5p mimics or inhibitors were co‐transfected with luciferase plasmids into 293 T cells, and after 48 h the luciferase activity was detected with a dual‐luciferase reporter kit (Beyotime, RG008), pRL‐TK with Renilla luciferase was the control plasmid (Wu et al., 2019).

### 2.4 MiRNA microarray

An Affymetrix miRNA microarray was used to profile the miRNAs in the exosomes released by IH A549 cells, RA A549 cells, and A549 cells under IH or RA (GSE number, 181067). The exosomes were isolated, as described above. The total RNA was extracted using a mirVana™ miRNA Isolation Kit (AM1561), and a miRNA microarray was performed by Boao Bio‐tech Inc. (Beijing, China). Briefly, the assay started with 8 μg of total RNA. After size fractionation of the RNAs using a YM‐100 Microcon centrifugal filter from Millipore, poly(A) tails were added to RNA sequences with lengths less than 300 nucleotides using poly(A) polymerase. An oligonucleotide tag was ligated to the poly(A) tail for later fluorescent dye staining. RNA samples were hybridized overnight on a Paraflo microfluidic chip using a micro‐circulation pump developed by Atactic Technologies. The probes were made in situ using photogenerated reagent chemistry. The hybridization melting temperatures were balanced by chemical modifications of the probes. Hybridization reactions were performed in 100 μL 6× SSPE buffer (0.90 M Sodium chloride, 60 mM Sodium hydrogen phosphate, 6 mM EDTA, pH 6.8) containing 25% formamide at 34°C. After RNA hybridization, tag‐conjugating Cy3 dyes (one‐color hybridization) were circulated to samples for dye staining. Each analyzed miRNA was repeated five times. A GenePix 4000B (Molecular Device, Union City, CA) laser scanner was used to collect the fluorescence images, which were digitized using Array‐Pro image analysis software (Media Cybernetics, Bethesda, MD).

### 2.5 Macrophage internalization of exosomes

Exosomes were labeled with the PKH26 kit (MKBio‐MX4021, Shanghai, China) according to a previous study (Bang et al., 2014). To summarize, IH and RA exosomes were diluted with PBS, PKH26 was added to incubate for 4 min. Labeled exosomes were incubated for 5 h with differentiated macrophages. The uptake of exosomes by macrophages was observed.

### 2.6 Cell transfection

Cell transfections were conducted according to a previous study (Zhang, Sai, et al., 2019). MiR‐106a‐5p mimics or inhibitors were synthesized by RiboBio Company (Guangzhou, China). The mRNA strands were as follows: MiR‐106a‐5p NC:TGTCACGTCTTCATAAATT, miR‐106a‐5p mimic: AAAAGTGCTTACAGTGCAGGTAG, miR‐106a‐5p inhibitor: TGTAAGCACTTCTTACATT. Differentiated macrophages were seeded into six‐well plates 24 h before transfection. When the cells reached 50% confluency, miR‐106a‐5p mimics (50 nM) or miR‐106a‐5p inhibitor (100 nM) were transfected into the cells with Lipofectamine 3000 (L3000‐015, Bjnobleryder, Beijing). Full‐length PTEN cDNA was synthesized using total RNA extracted from A549 cells as a template. The following primers were used: PTEN, forward: 5‐CCGCTCGAGATGACAGCCATCATCAAAG‐3 and reverse: 5‐CGCGGATCCTCAGACTTTTGTAATTTGTG3. The cDNA was then cloned into the pcDNA3.1(−) expression vector (Invitrogen) using Lipofectamine® 2000. Empty pcDNA3.1(−) was used as a control. PTEN expressing plasmid was amplified via PCR and cloned into the vector (Xiong et al., 2018).

### 2.7 RNA interference

The siRNA sequence of STAT3 was synthesized by GeneCreate Biological Engineering Co., Ltd (Wuhan, China). The STAT3 siRNA sense was CCAGGCACCTTCCTGCTAA, and the siRNA NC sense was: GCATAGACCGTCGTCAATT. 100 nM of STAT3 siRNA was transfected into macrophages, using Lipofectamine™ 2000 transfection reagent (Thermo‐Life, Shanghai, China).

### 2.8 Cck8 assay

A549/NCI‐H226 cells were seeded in 96‐well plates (2 × 10^3^ cells/well) and cultured with 50 μL macrophage culture medium mixed with 50 μL ordinary medium for 48 h. And on the third day, change the same culture medium again till the fifth day. CCK8 solution reagent (10 μL) (Biyuntian Biotechnology Co., LTD, C0038) was added to each well. After a 2 h incubation at 37°C with 5% CO_2_ in an incubator, cell proliferation was measured using a 96‐well plate reader.

### 2.9 Migration assay

For the migration assay, 5 × 10^4^ A549/NCI‐H226 cells were seeded on the top chamber of 24 wells transwell chambers (Corning Inc., 3422) with 8‐μm inserts. The cell culture media from the macrophages were added to the bottom chamber. After 24 h incubation for A549/NCI‐H226 cells, the cells that migrated through the membrane were fixed in methanol and stained with 1% crystal violet (Beyotime Biotechnology, C0121). The cells were washed with PBS to remove excess dye, and the non‐cellular inoculation side was photographed under a microscope (IX71, Olympus, Japan). All experiments were performed three or more times.

### 2.10 Quantitative real‐time PCR

The procedure used for PCR has been previously described (Ren et al., 2017). Briefly, total RNA was extracted from the exosomes or the cells, total RNA was reverse transcribed into cDNA, and PCR was performed using SYBR GREEN. The primer was synthesized by Wuhan Jinkairui Biological Engineering Co., LTD. The following primers were used: U6 primers, forward: CTCGCTTCGGCAGCACAT, reverse: AACGCTTCACGAATTTGCGT; hsa‐miR‐106a‐5p primers, forward: TGGGTGCTTACAGTGCAGGTAG, reverse: CTCAACTGGTGTCGTGGAGTC; GAPDH primers, forward: CATCATCCCTGCCTCTACTGG, reverse: GTGGGTGTCGCTGTTGAAGTC; Arginase‐1 primers, forward: AGACCACAGTTTGGCAATTGG, reverse: AGGAGAATCCTGGCACATCG; iNOS primers, forward: GCAGGACTCACAGCCTTTGG, reverse: GGCTGGATGTCGGACTTTGT; IL‐1β primers, forward: ACGATGCACCTGTACGATCACT, reverse: GAGAACACCACTTGTTGCTCCA; CD206 primers, forward: GATGATACCTGCGACAGTAAACG, reverse: GCTTGCAGTATGTCTCCGCTT; CD163 primers, forward: TGGAGTTGCCCTTTCTACCC, reverse: CCTCCATTTACCAGGCGAAG; IL‐10 primers, forward: AACCTGCCTAACATGCTTCG, reverse: GAGTTCACATGCGCCTTGAT; TGFβ1 primers, forward: CAGCAACAATTCCTGGCGATA, reverse: GCTAAGGCGAAAGCCCTCAAT; PTEN primers, forward: ATTCCCAGTCAGAGGCGCTAT, reverse: GAACTTGTCTTCCCGTCGTGT.

PCR was performed using the following conditions: the cycling profile started with denaturation at 95°C for 30 s, followed by 40 cycles of 5 s at 95°C and 34 s at 60°C. For each gene, “no‐tempalte” and “no‐amplification” controls were used. Subsequently, a melting curve analysis was performed to measure the specificity of the amplified products by their specific melting temperatures. Melting curve analysis consisted of a denaturation step at 95°C for 15 s, after which the reactions were decreased to 60°C for 1 min and then increased to 95°C for 15 s. Data for each sample were normalized against GAPDH, all experiments were performed three or more times.

### 2.11 Western blot

The Western blot procedure has been previously described (Ren et al., 2018). The proteins were separated by 10% SDS‐PAGE, then transferred onto PVDF membranes and blotted with specific antibodies at 4°C overnight, and then incubated with the horseradish peroxidase‐conjugated secondary antibody (1:5000) at 37°C for 1 h. They were then subjected to chemiluminescent detection according to the manufacturer's instructions (Santa Cruz Biotechnology, USA). All bands were evaluated by densitometry with Quantity One V4.6.2 software (Bio‐Rad, USA). Bands of interest were normalized against GAPDH. The data are presented as relative density ratios. The membranes were incubated overnight at 4°C with the following primary antibodies: anti‐CD9 (Abcam, ab92726), anti‐IL‐1β (Abcam, ab9722), anti‐CD63 (Lsbio, LS‐C408817), anti‐HSP70(Wuhan Sanying Biotechnology Co., LTD,10995‐1‐AP), anti‐PTEN (Abcam, ab170941), anti‐iNOS (CST, #32027), anti‐CD206 (Abcam, ab125028), anti‐CD163 (Abcam, ab182422), anti‐TGFβ1(Wuhan Sanying Biotechnology Co., LTD,21898‐1‐AP), and anti‐IL‐10 (Bioss, bs‐0698R); anti‐N‐Cadherin (CST, 4061), anti‐Vimentin (Abcam, ab92547), anti‐MMP‐7 (Abcam, ab189277), anti‐CD81 (Abcam, ab109201), anti‐TSG101 (Abcam, ab125011), anti‐STAT3 (Abcam, ab68153), anti‐IL‐6(Wuhan Sanying Biotechnology Co., LTD, 21865‐1‐AP), anti‐CXCR4 (Abcam, ab124824), anti‐Arginase‐1 (Santa, sc‐47,715), anti‐E‐Cadherin (CST, #14472), and anti‐CCL2 (Abcam, Cambridge, MA, USA); and anti‐GAPDH (Sigma‐Aldrich, St. Louis, MO, USA). Followed by a second incubation at room temperature with the secondary antibody (ASPEN, AS1107) containing horseradish peroxidase. Images were analyzed by ImageJ software, and all experiments were performed three or more times.

### 2.12 Animal experiments

Male BALB/c nude mice (4 weeks old) were purchased from Charles River Laboratories (Beijing, China). All animal experiments were approved by the Institutional Animal Care and Use Committee (IACUC). 100 μL A549 cells (5 × 10^6^cells/200 μL), 100 μL macrophages (5 × 10^6^cells/200 μL) or 100 μL exosomes (1 × 10^6^ exosomes/100 μL) were injected subcutaneously into the back of nude mice. Tumor growth was followed for 5 weeks from the first injection. Tumor volume was measured once a week with calipers and calculated according to the formula V = 0.5*W* ^*2*^ *L* (W = tumor width; (L) = tumor length). Five weeks later, mice were euthanized with 10 mg/mL of pentobarbital solution by intraperitoneal injection. Tumors were carefully excised, weighed, and immediately frozen at −80°C for future use.

### 2.13 Preparation of purified macrophages in tumor tissue of mice

Macrophages were prepared as previously report (Kang et al., 2017). The tumor tissue was passed through 00 wire mesh to isolate individual cells. The following processes are carried out at 4°C. Centrifuge 200 × *g* for 10 min and remove the supernatant. The test tube was lightly turned, the cells were suspended, 10 mL hypoexudative solution (20 mmol/L Tris, 0.75% ammonium chloride, pH 7.4) was added, and treated at 37°C for 3–5 min to dissolve the red blood cells. Centrifuge 200 × *g* for 10 min and remove the supernatant. The cells were washed once with RPMI‐1640 culture solution. Cell suspension was added to lymphocyte isolation solution (specific gravity 1.077), centrifuged 400 × *g* for 20 min, and interface macrophages were collected. Discard precipitated dead cells and red blood cells. Macrophages were washed twice with RPMI‐1640 culture solution. Trypan blue staining was used to detect cell viability and cell number, and the cells were matched to the required concentration. At this time, macrophage vitality can reach more than 90%, and macrophages account for more than 90% of all cells.

### 2.14 Human clinical samples

From May 1, 2020, to May 1, 2022, 50 patients with NSCLC complicated with OSAS who never received any anti‐cancer therapy (radiotherapy, chemotherapy, or biotherapy) or CPAP treatment were enrolled. The TNM stage of the patients with NSCLC were phase II/III. Blood samples were centrifuged at 3000 × *g* for 15 min at 4°C. The serum was collected in a fresh tube and centrifuged at 12,000 × *g* for 10 min at 4°C to remove cellular fractions. The expression of miR‐106a‐5p in the serum exosomes of each group was detected. The clinicopathological characteristics of the patients are summarized in Table 1. The median was used as a cut‐of value which defined the high and low expression of miR‐106a‐5p. The peripheral blood samples from patients and health volunteers were approved by the Institutional Review Board of the First Affiliated Hospital of Zhengzhou University (approval number: 2020‐KS‐HNSR188). Written informed consent was obtained from all study participants or their guardians.

**TABLE 1:** Correlation between miR‐106a‐5p expression and clinicopathological parameters in patients with OSA complicated with stage II/III non‐small cell lung cancer.

Parameters	Patient number	miR‐106a‐5p expression		*p* value
	*n* = 50	Low	High	
Age (years)				0.2545
≥60	28	16	12	
<60	22	9	13	
Gender				0.3946
Male	25	15	10	
Female	25	12	13	
AHI				<0.001
5 ≤ AHI<15 times/h	14	13	1	
15 ≤ AHI<30 times/h	18	11	7	
AHI≥30 times/h	18	2	16	
Pathological type				0.2575
Squamous carcinoma	25	11	14	
Adenocarcinoma	25	15	10	

*Note*: For analysis of correlation between miR‐106a‐5p expression and clinical features, Fisher exact tests were used. The median was used as a cut‐of value which defined the high and low expression of miR‐106a‐5p.

### 2.15 Flow cytometry

In this study, flow cytometric analysis was used to detect the immunophenotype of macrophages in tumor tissues of xenografted tumors in mice or peripheral blood with GCMSCs. Briefly, a single‐cell suspension of macrophages was first incubated with CD206‐FITC (human), CD206‐PE (mouse) for 30 min at 4°C in the dark. After washing, the labeled cells were resuspended in PBS and analyzed on a FACSCanto II flow cytometer (BD Biosciences, Sparks, MD, USA). As negative controls, isotype‐matched antibodies with the corresponding fluorescent labels were used (Cheng et al., 2018). All experiments were performed three or more times.

### 2.16 Statistical analysis

The data are expressed as the means ± standard deviation (SD). Student's *t* test (for comparisons between two groups) and one‐way NOVA (for comparisons among three or more groups). The statistical analysis was conducted and visualized using the GraphPad Prism Software. The values of *p* < 0.05 were considered significant.

## 3 RESULTS

### 3.1 Exosomes derived from IH A549 cells promote M2 macrophage polarization

**FIGURE 1:**
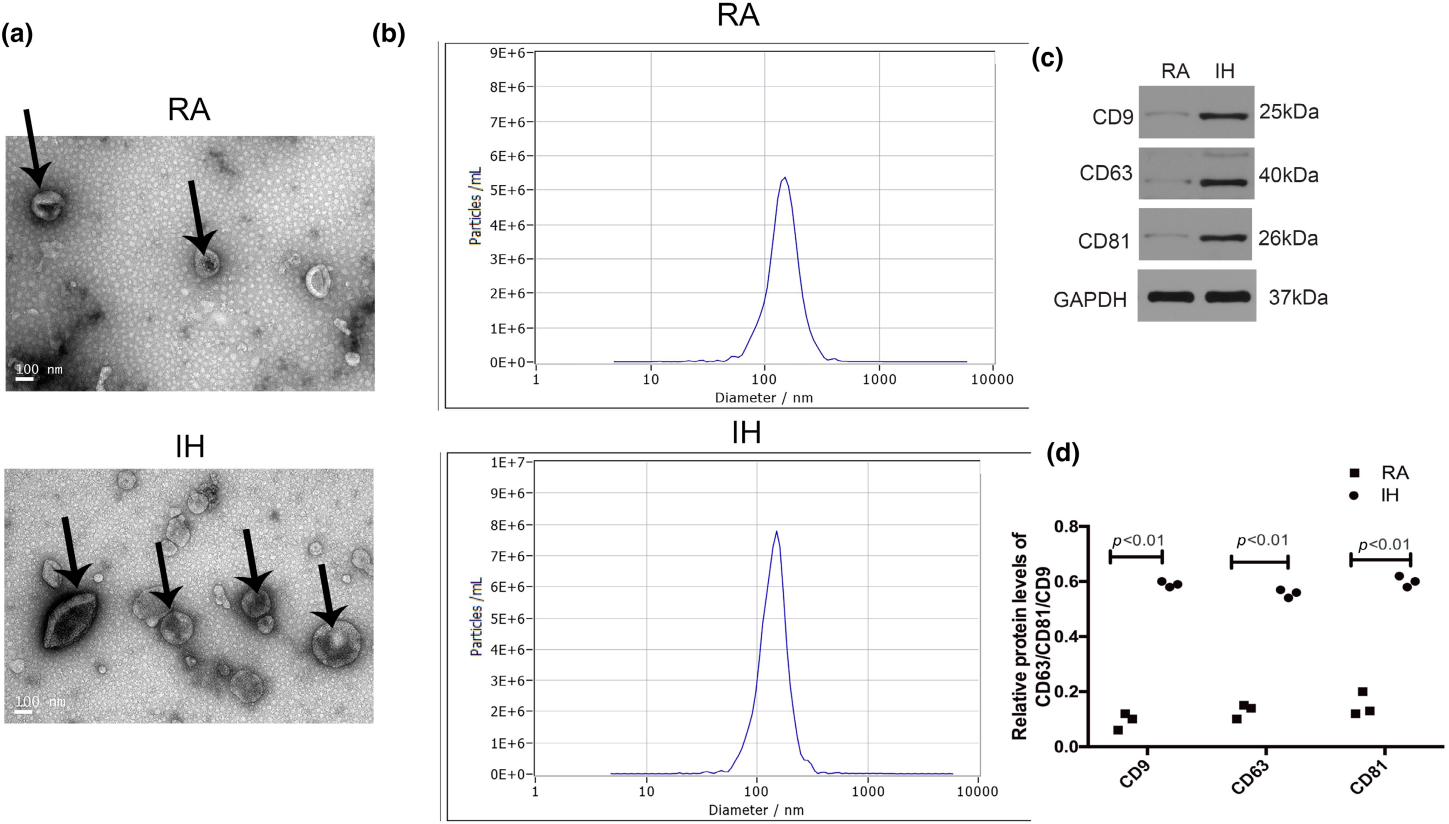
Exosomes from IH and RA conditions. (a) Electron microscope image of exosomes derived from A549 cells. (b) Exosome concentration for nanoparticle tracking analysis. *n* = 5. (c) The protein expression level of exosome markers. All data are expressed as the means ± SD. (d) Densitometric evaluation of western blotting results. *n* = 3.

In order to detect the effect of IH and RA on exosomes secreted by A549 cells, we cultured A549 cells under IH or RA conditions for 48 hours and then extracted the exosomes from the culture medium for identification. As shown in Figure 1a, the electron microscope showed that the diameters of the purified particles were between 50 and 120 nm, and the nanoparticle tracking analysis showed the size distribution and concentration of the exosomes. The exosome concentration of A549 cells in the IH group was higher than that in the RA group (Figure 1b). The western blot analysis of exosomal proteins showed that the expressions of CD9, CD63, and HSP70 in A549 cells were increased after IH exposure (Figure 1c,d). This data demonstrated that IH increased the secretion of exosomes by A549 cells.

**FIGURE 2:**
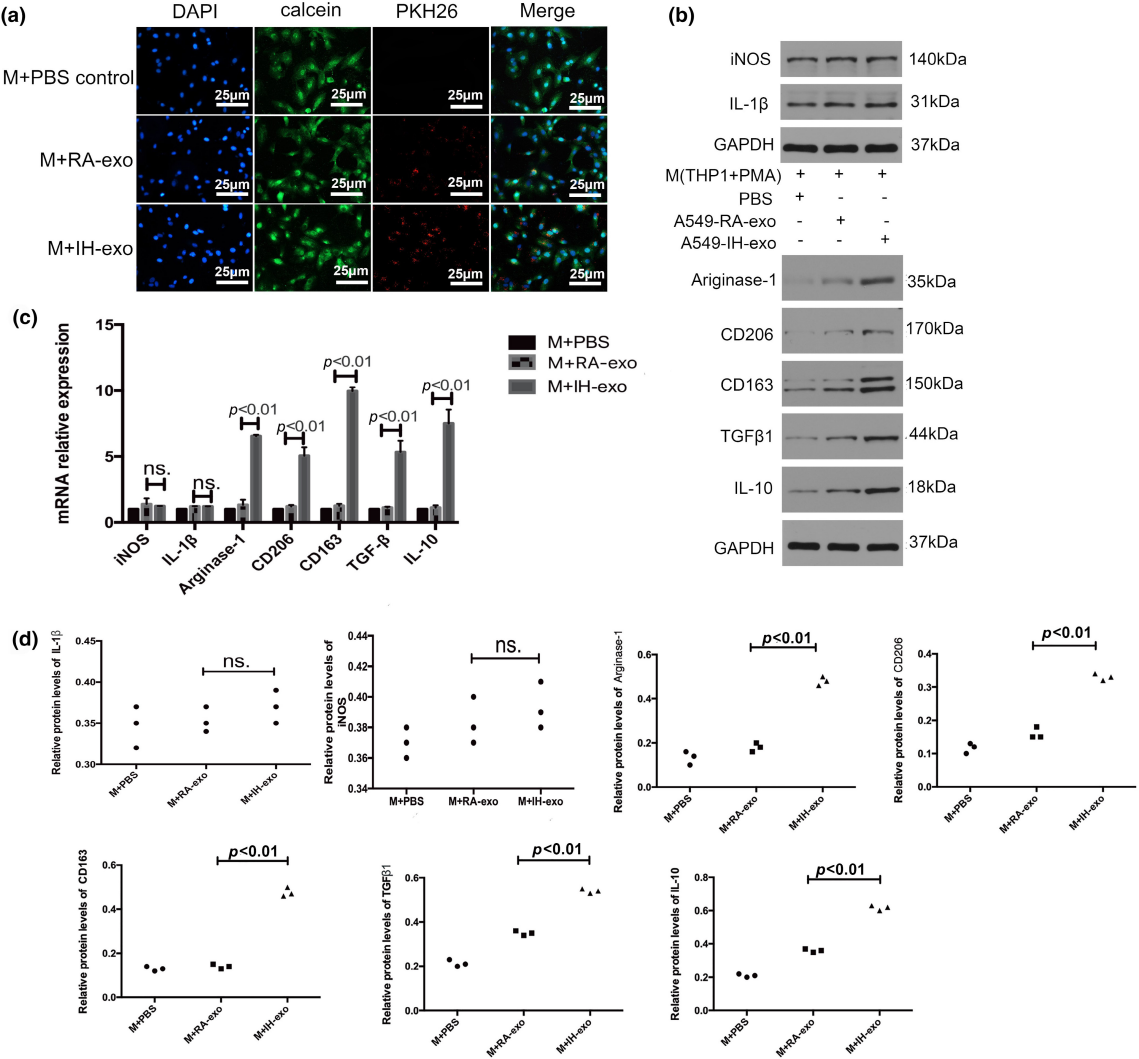
PKH26 labeled exosomes entered into macrophages and promoted the polarization of macrophages toward M2‐type. (a) Representative images were filmed after cells were fixed and stained (magnification, 400×). (b–d) mRNA and protein expression levels of M1 and M2 markers in macrophages after incubation of PBS, RA exosomes and IH exosomes. *n* = 3.

We then conducted exosome uptake experiments. The exosomes were labeled with PKH26 (Sigma, USA) and were incubated with macrophages. As shown in Figure 2a, the fluorescent exosomes entered the macrophages. The expression levels of M2 macrophage markers (including CD206, CD163, IL‐10, TGF‐β1, and Arginase‐1) were higher in the macrophages incubated with the IH exosomes in comparison to the RA exosomes and PBS. There were no significant differences in the expression levels of M1 macrophage markers (including iNOS and IL‐1) among the three groups (Figure 2b–d).

### 3.2 Macrophages cultured with IH exosomes promote the proliferation, migration, and invasion of NSCLC cells

**FIGURE 3:**
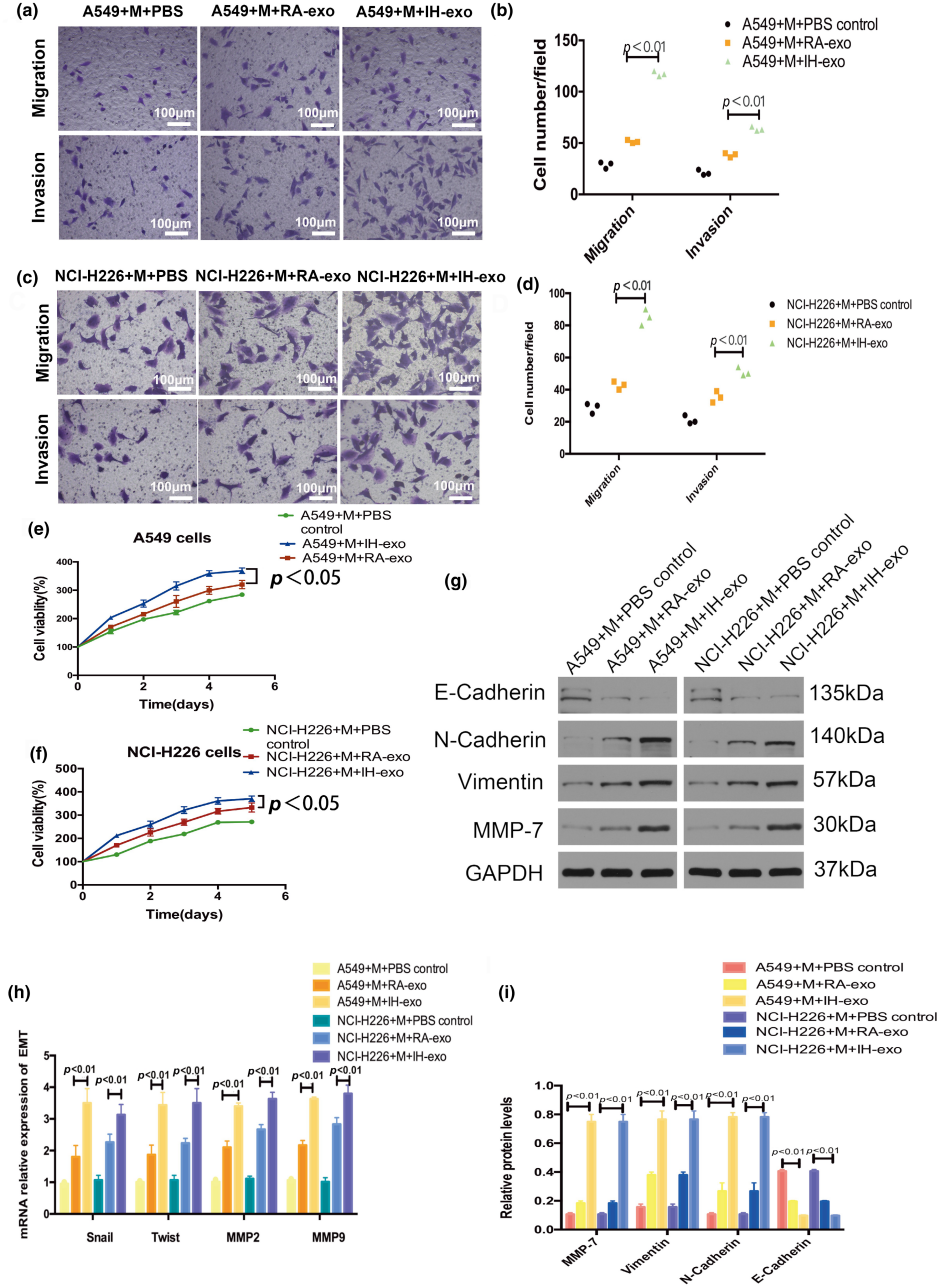
IH exosomes promoted NSCLC via EMT. (a–d) Cell migration and invasion were measured by transwell assays. (e, f) The proliferation ability of NSCLC cells were treated with macrophages cocultured with IH A549‐secreted exosomes, RA A549‐secreted exosomes or PBS, (g, i) the epithelial and mesenchymal markers in NSCLC cells were measured by western blot, (h) Snail, Twist, MMP2, and MMP9 were measured by PCR. *n* = 3.

To further investigate whether macrophages educated by IH exosomes have the characteristic function of tumor promotion, we collected the supernatant from macrophages co‐cultured with RA exosomes, IH exosomes or PBS. The A549 and NCI‐H226 cells were treated with different conditioned media of macrophages for 5 days, and then CCK8 assays were performed daily to assess proliferation, cellular migration and invasion was evaluated by a migration and invasion transwell assay. As shown in the results, conditioned media from macrophages treated with IH exosomes promoted the proliferation, migration and invasion abilities of A549 and NCI‐H226 cells compared to macrophages treated with RA exosomes or macrophages treated with PBS (Figure 3a–d).

We then investigated whether IH exosomes had an effect on epithelial‐mesenchymal transformation (EMT). As shown in Figure 3e–i, the mesenchymal cell markers (N‐cadherin, Vimentin, MMP7, Snail, Twist, MMP2, and MMP9) were higher and the epithelial marker E‐cadherin was lower after treatment with macrophages treated with IH exosomes compared to the macrophages treated with RA exosomes or macrophages treated with PBS.

### 3.3 MiR‐106a‐5p was highly expressed in exosomes derived from IH A549 cells and could regulate the expression of PTEN

**FIGURE 4:**
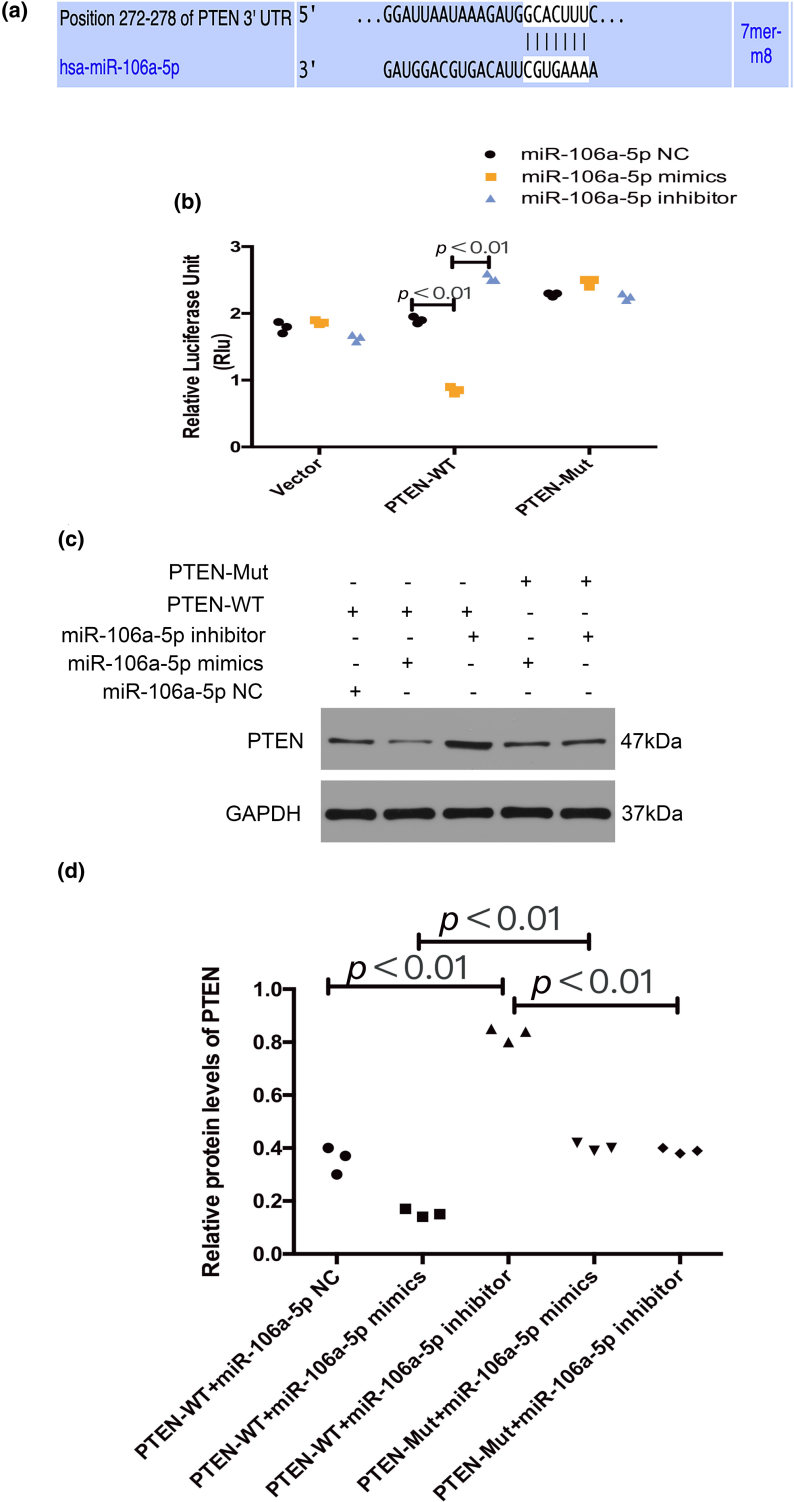
Regulation of PTEN expression by miR‐106a‐5p. MiR‐106a‐5p could regulate the expression of PTEN. (a) The binding site of miR‐106a‐5p on PTEN. (b) Dual‐luciferase reporter gene assay was used to verify the targeting relationship between miR‐106a‐5p and PTEN. (c, d) The expression of PTEN was detected by western blot after miR‐106a‐5p was overexpressed or inhibited in macrophages. *n* = 3.

It has been widely accepted that miRNAs in exosomes mediate intercellular communication; thus, we used an Affymetrix miRNA microarray to profile the miRNAs in IH A549‐derived exosomes and RA A549‐derived exosomes, miRNAs in IH A549 cells and RA A549 cells. Compared to RA A549‐derived exosomes, 2 miRNAs were up‐regulated in IH A549‐derived exosomes. Compared to RA A549 cells, 84 miRNAs were up‐regulated in IH A549 cells. The differential expression of the thermal map from the chips were plotted (GSE181067, https://www.ncbi.nlm.nih.gov/geo/info/linking.html). Then we identified the overlap of shared exosomal miRNAs and miRNAs in A549 cells. Two miRNAs including has‐miR‐1231 and has‐miR‐106a‐5p were up‐regulated in the IH group compared to the RA group. Through literature review, we found that miR‐106a‐5p expression was increased in NSCLC (Shan et al., 2018). In order to further explore the mechanism by which exosomes promote tumor development, we used miRNA‐BASE to predict the target genes of miR‐106a‐5p. Among them was PTEN, a significant tumor suppressor gene, and loss of PTEN function can have a dramatic impact on tumorigenesis and cancer progression (Carracedo et al., 2011). The binding site of miR‐106a‐5p on PTEN is shown in Figure 4a. Dual‐luciferase reporter displayed that miR‐106a‐5p mimics were capable of repressing the luciferase activity of wild type PTEN reporter, but miR‐106a‐5p mimics didn't change the luciferase activity of the mutant PTEN reporter (Figure 4b). Western blot assay showed that miR‐106a‐5p mimics inhibited the expression of wild‐type PTEN, while inhibition of miR‐106a‐5p increased the expression of wild‐type PTEN, no changes were found in mutant PTEN (Figure 4c,d). As such, miR‐106a‐5p and its target gene PTEN are clearly involved in the tumor‐related signaling pathways and were selected as the subjects of the next experiment.

### 3.4 miR‐106a‐5p is secreted by IH lung cancer cells and transferred to macrophages via exosome secretion

**FIGURE 5:**
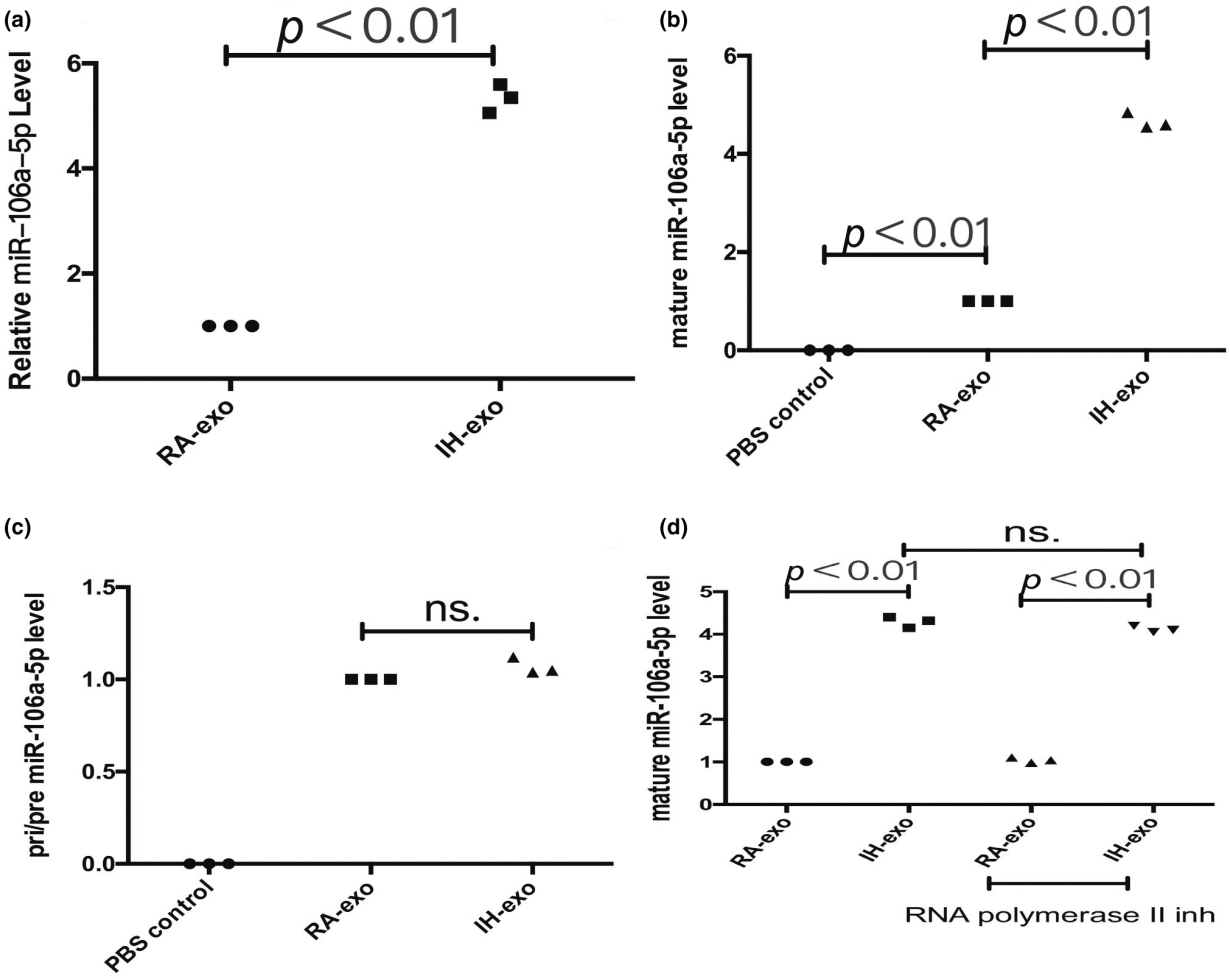
The transfer of exosomal miR‐106a‐5p isolated from IH A549 cells to macrophages. (a) The level of miR‐106a‐5p in exosomes of A549 cells lines under RA and IH conditions. The level of mature (b) and pri/pre miR‐106a‐5p (c) in exosome‐treated macrophages. (d) RNA polymerase II inhibitor did not change the level of miR‐106a‐5p in macrophages exposed to IH A549‐dervied exosomes. *n* = 3. ns, no significant difference.

To confirm that miR‐106a‐5p can be transferred to macrophages via exosomes, we determined miR‐106a‐5p levels in macrophages treated with the exosomes isolated from A549 cells under RA and IH conditions (Figure 5a). An increase in cellular level of mature miR‐106a‐5p, but not pri‐/pre‐miR‐106a‐5p, was observed in recipient macrophages following the treatment with exosomes derived from IH A549 cells (Figure 5b,c). In addition, the increase of miR‐106a‐5p in macrophages cultured with exosomes derived from IH A549 cells was not prevented by an RNA polymerase II inhibitor (Figure 5d). These data reveal that IH exosomes containing miR‐106a‐5p were internalized by macrophages.

### 3.5 Exosomal miR‐106a‐5p promotes the proliferation, invasion and metastasis of A549 cells by polarizing macrophages

**FIGURE 6:**
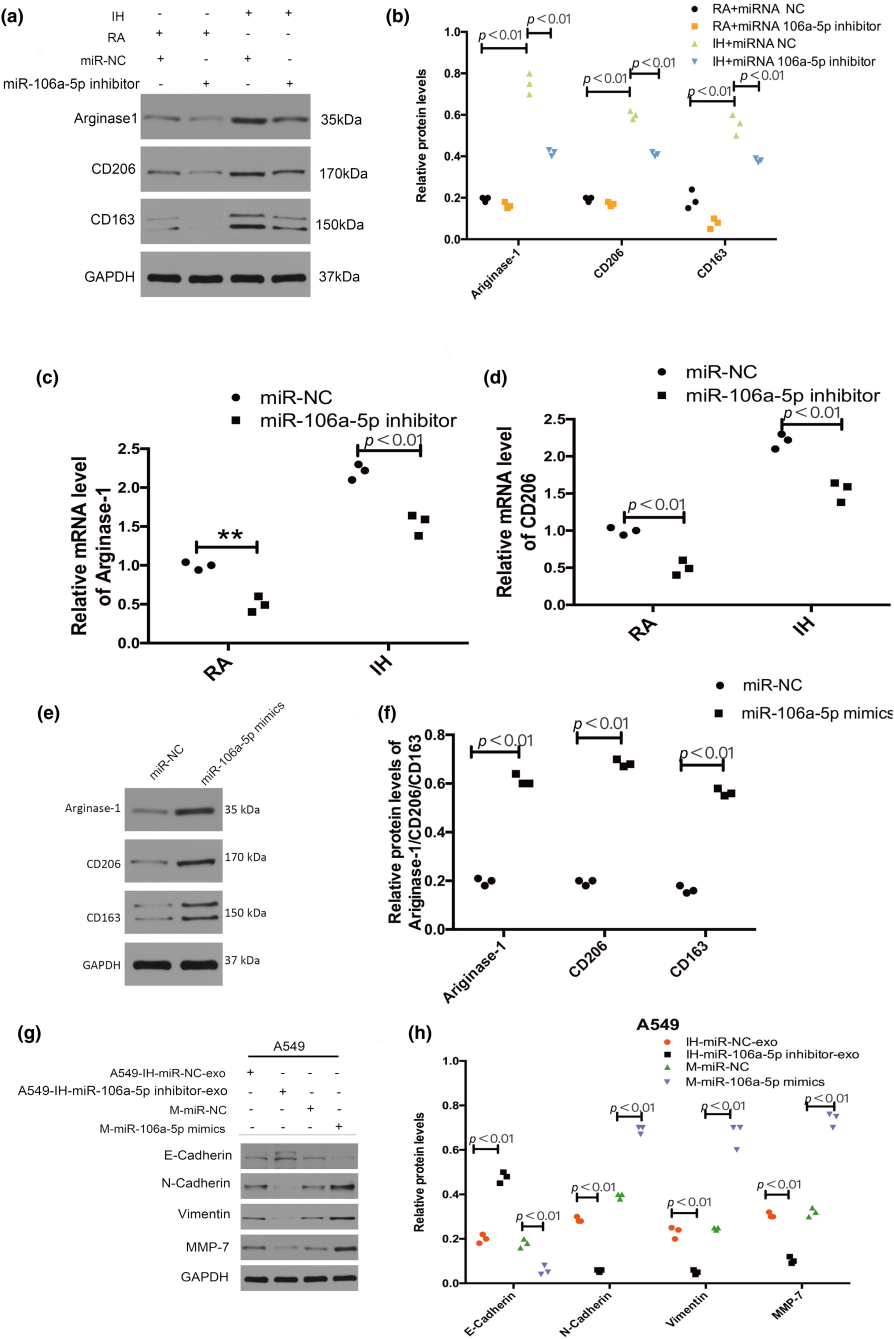
Exosomal miR‐106a‐5p promotes NSCLC by polarizing macrophages. (a–d) The expression of M2 macrophage markers when macrophages were incubated with exosomes isolated from miR‐106a‐5p inhibited A549 cells or miR‐106a‐5p NC A549 cells. (e, f) The expression of M2 macrophage markers when macrophages were transfected with miR‐106a‐5p mimics. (g, h) The expression levels of epithelial cell marker (E‐cadherin) and mesenchymal cell markers (N‐cadherin, Vimentin, MMP7) in the A549 cells when they were treated with macrophages transfected with miR‐106a‐5p mimics or miR‐106a‐5p NC, macrophages treated with IH miR‐106a‐5p inhibited A549‐derived exosomes or IH miR‐106a‐5p NC A549‐derived exosomes. *n* = 3.

Inhibition of miR‐106a‐5p in A549 cells by miR‐106a‐5p inhibitor transfection abolished the effect of IH A549‐derived exosomes on M2 macrophage markers (CD206, CD163, and Arginase‐1) upregulation (Figure 6a–d). Transfection with miR‐106a‐5p mimics increased the expression level of M2 macrophage markers (CD206, CD163, and Arginase‐1) in macrophages (Figure 6e,f). And macrophages transfected with miR‐106a‐5p mimics had decreased expression levels of epithelial cell marker (E‐cadherin), and increased expression levels of mesenchymal cell markers (N‐cadherin, Vimentin, and MMP7) in A549 cells. Macrophages treated with IH miR‐106a‐5p inhibited A549‐derived exosomes decreased the expression of mesenchymal cell markers (N‐cadherin, Vimentin, and MMP7) and increased the expression of epithelial cell marker (E‐cadherin) in A549 cells (Figure 6g,h).

**FIGURE 7:**
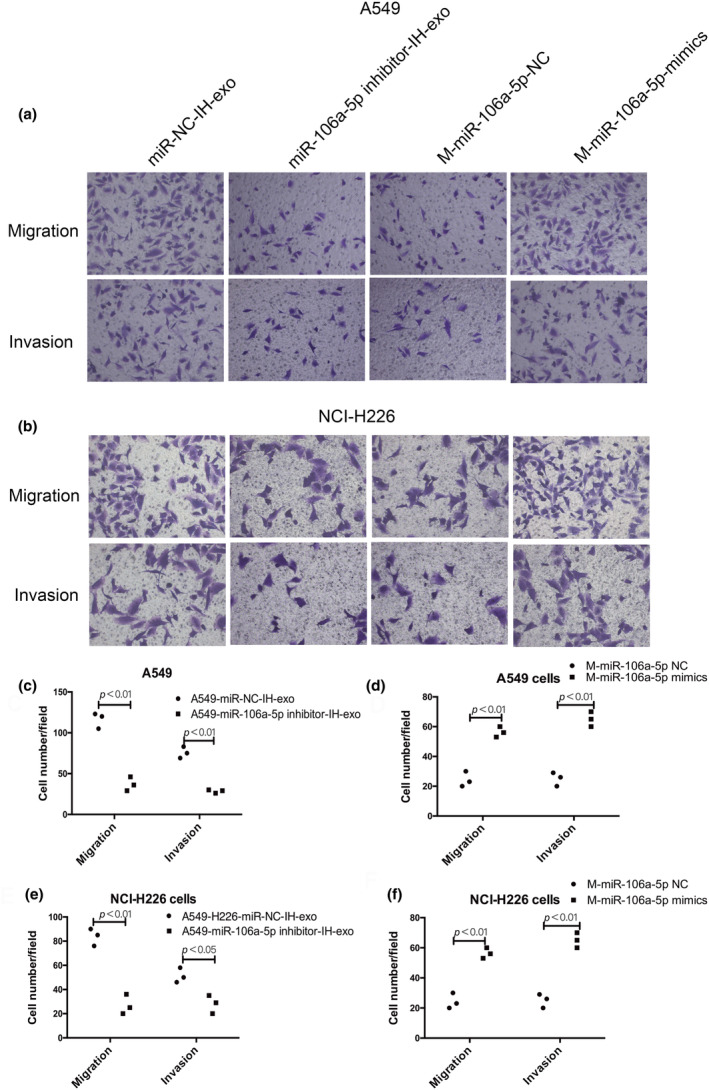
Exosomal miR‐106a‐5p promotes NSCLC by polarizing macrophages. The ability for migration and invasion of A549 cells (a, c, d) and NCI‐H226 cells (b, e, f) were decreased by the macrophages treated with the exosomes from IH miR‐106a‐5p inhibited A549 cells and were increased by macrophages transfected with miR‐106a‐5p mimics. *n* = 3.

The ability for migration and invasion of the A549 and NCI‐H226 cells were decreased by the macrophages treated with the exosomes isolated from IH miR‐106a‐5p inhibited A549 cells compared to macrophages treated with the exosomes isolated from IH miR‐106a‐5p NC A549 cells, and macrophages transfected with miR‐106a‐5p mimics promoted the migration and invasion of lung cancer cells compared to macrophages transfected with miR‐106a‐5p NC (Figure 7).

### 3.6 Exosomal miR‐106a‐5p induced the polarization of M2 macrophages by inhibiting the PTEN or activating or STAT3 signaling pathway

**FIGURE 8:**
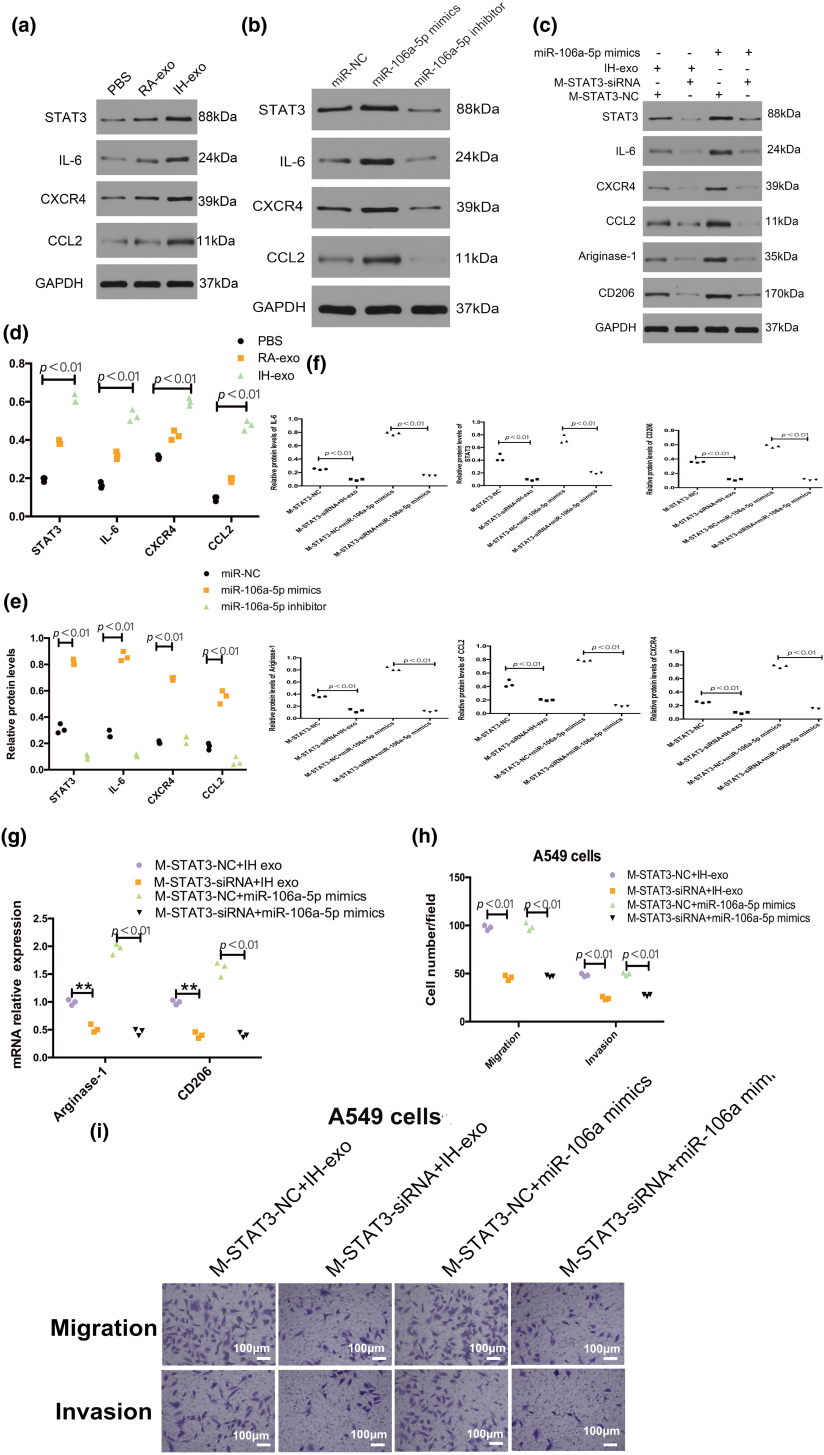
Role of the STAT3 signaling pathway in the migration and invasion ability of A549 cells and macrophages. (a–g) The expression of STAT3, IL‐6, CXCR4, and CCL2 and M2 macrophage markers (CD206 and Arginase‐1) in macrophages. (h, i) The ability of migration and invasion of A549 cells were increased by IH exosomes or macrophages transfected with miR‐106a‐5p mimics, but were down‐regulated by STAT3 knockdown. *n* = 3.

Compared with the RA exosomes, IH exosomes could increase the expression of STAT3, IL‐6, CXCR4 and CCL2 in macrophages (Figure 8a,d), and transfection with miR‐106a‐5p mimics significantly increased the expression of STAT3, IL‐6, CXCR4 and CCL2 in macrophages, however, inhibition of miR‐106a‐5p exhibited the opposite effect (Figure 8b,e). To explore whether exosomal miR‐106a‐5p promoted macrophage polarization through the STAT3 signaling pathway, we further explored the mechanism by transfecting macrophages with STAT3 siRNA. As shown in Figure 8c,f, inhibition of STAT3 in macrophages by STAT3 siRNA transfection abolished the effect of IH exosomes on the expression of STAT3, IL‐6, CXCR4, CCL2 and M2 macrophage markers (CD206 and Arginase‐1) upregulation. Moreover, both the migration and invasion abilities of A549 cells co‐cultured with macrophages transfected with STAT3 siRNA were decreased regardless if the macrophages were treated with IH exosomes or transfected with miR‐106a‐5p mimics (Figure 8g–i). Together, this in vitro data indicates that IH exosomal miR‐106a‐5p induced the polarization of M2 macrophages through activation of the STAT3 signaling pathway.

**FIGURE 9:**
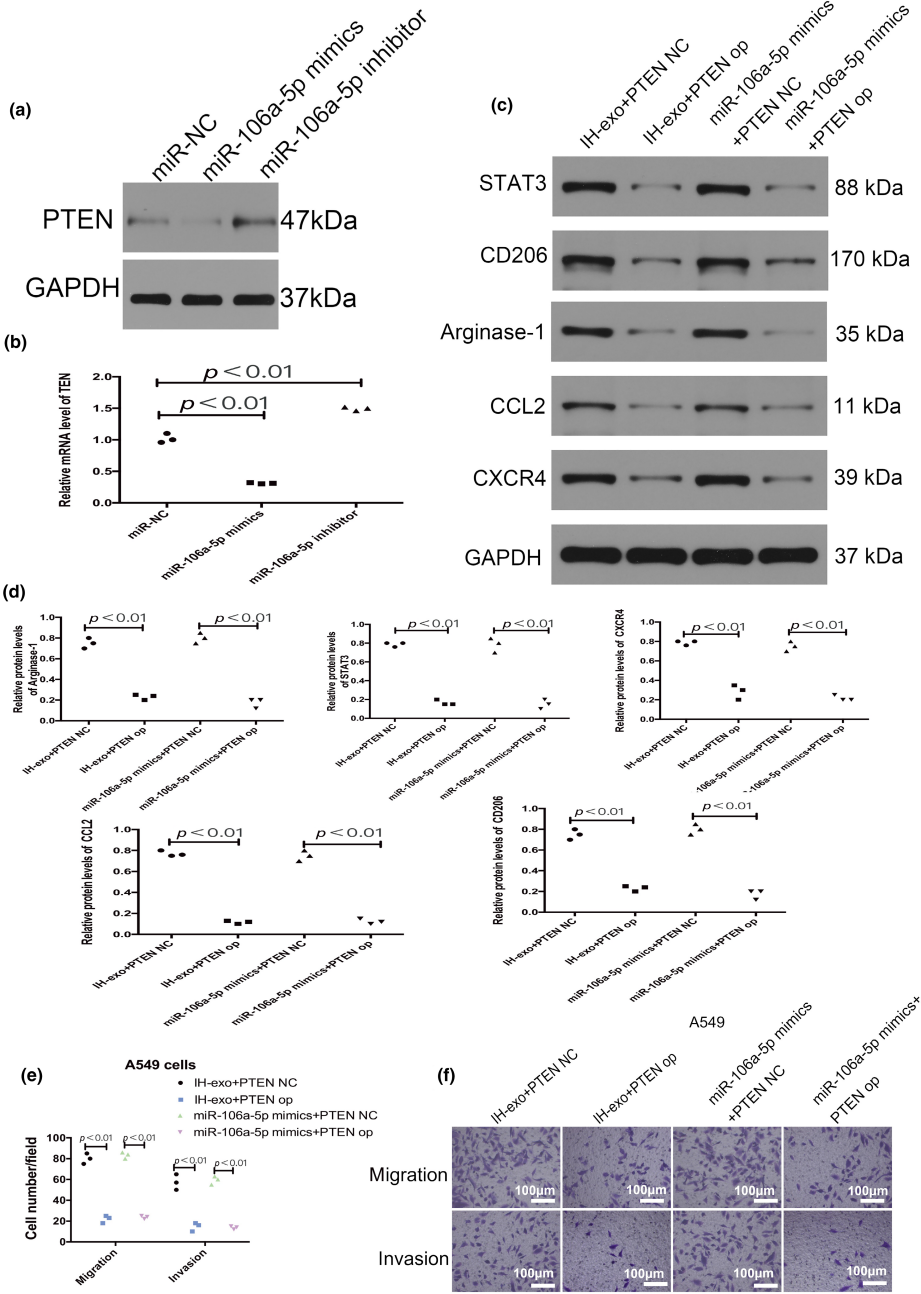
Role of PTEN in the migration and invasion ability of A549 cells and macrophages. (a, b) The expression of PTEN was decreased by miR‐106a‐5p mimics, and was increased by miR‐106a‐5p inhibitor. (c, d) The expression of STAT3, IL‐6, CXCR4, and CCL2 and M2 macrophage markers (CD206 and Arginase‐1) in macrophages. (e, f) The ability of migration and invasion of A549 cells. op, overexpression. *n* = 3.

Compared with the miR‐NC, miR‐106a‐5p mimics could decrease the expression of PTEN, but miR‐106a‐5p inhibitor had the opposite effect (Figure 9a,b). The results revealed IH exosomes or miR‐106a‐5p mimics induced the upregulation of M2 macrophage markers (CD206, and Arginase‐1), STAT3, CXCR4, and CCL2 in macrophages were reversed by overexpression of PTEN (Figure 9c,d). Moreover, transfected with the PTEN vector in macrophages abolished the promotion of migration and invasion abilities of A549 cells by the macrophages treated with IH exosomes or transfected with miR‐106a‐5p mimics (Figure 9e,f).

### 3.7 IH promotes the development of NSCLC by promoting macrophage polarization through exosomal miR‐106a‐5p in vivo

**FIGURE 10:**
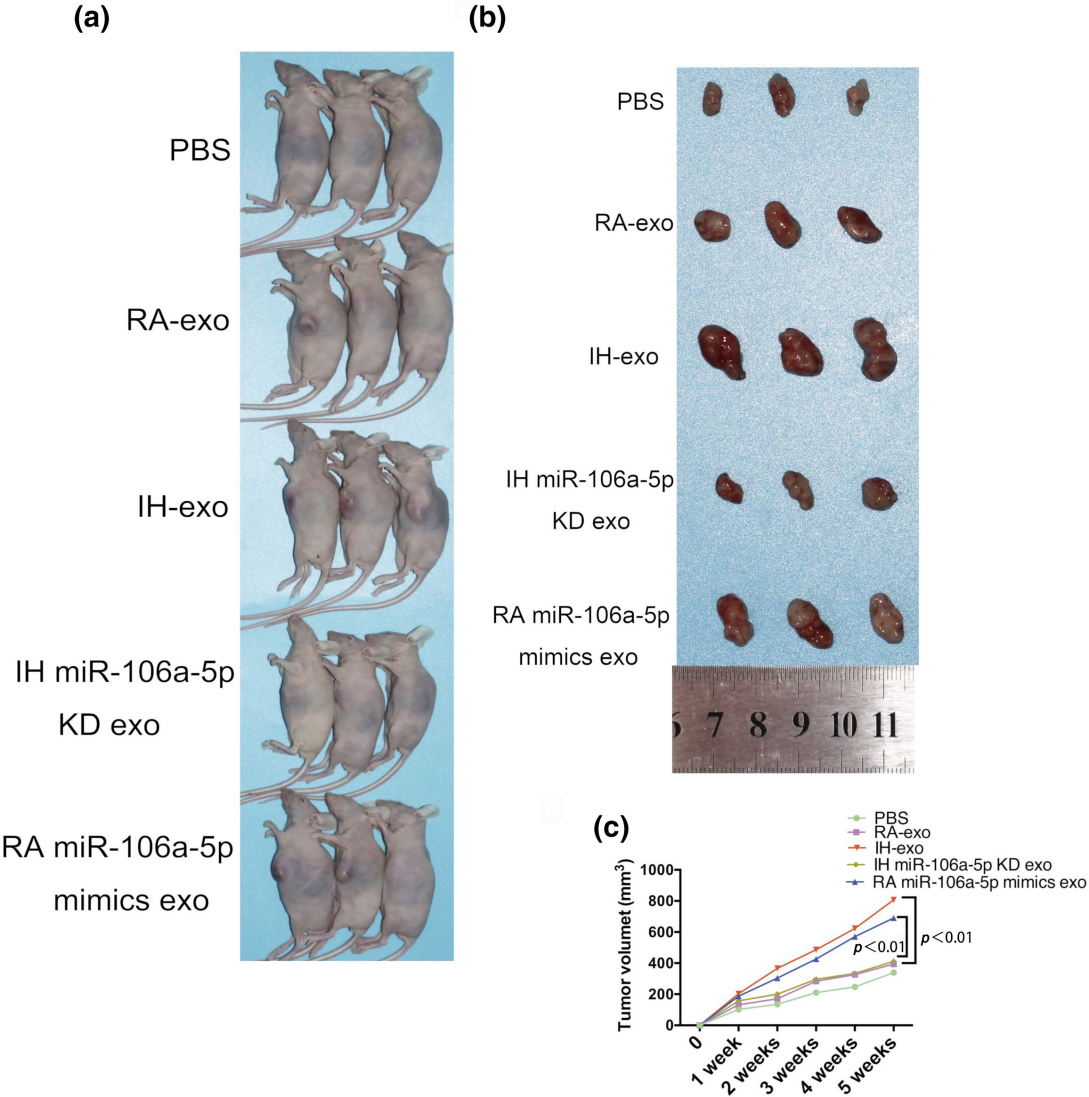
The role of IH A549‐derived exosomal miR‐106a‐5p in tumor growth in a xenograft model. (a, b) The photos of the nude mice. (c) Growth curve of xenograft tumors. KD, knockdown. *n* = 3.

Next, we used a mouse tumor model to evaluate whether exosomal miR‐106a‐5p promotes the growth of tumors in vivo. We injected A549 cells subcutaneously into the flanks of nude mice, macrophages co‐cultured with PBS, RA, and IH exosomes, exosomes isolated from RA miR‐106a‐5p mimic A549 cells, exosomes isolated from IH miR‐106a‐5p inhibited A549 cells were injected intratumorally, and the mass volume was measured once a week. The overexpression of miR‐106a‐5p in RA exosomes increased the growth of the tumor. Knockdown of miR‐106a‐5p abrogated the promotion of tumor growth by IH exosomes (Figure 10a–c).

**FIGURE 11:**
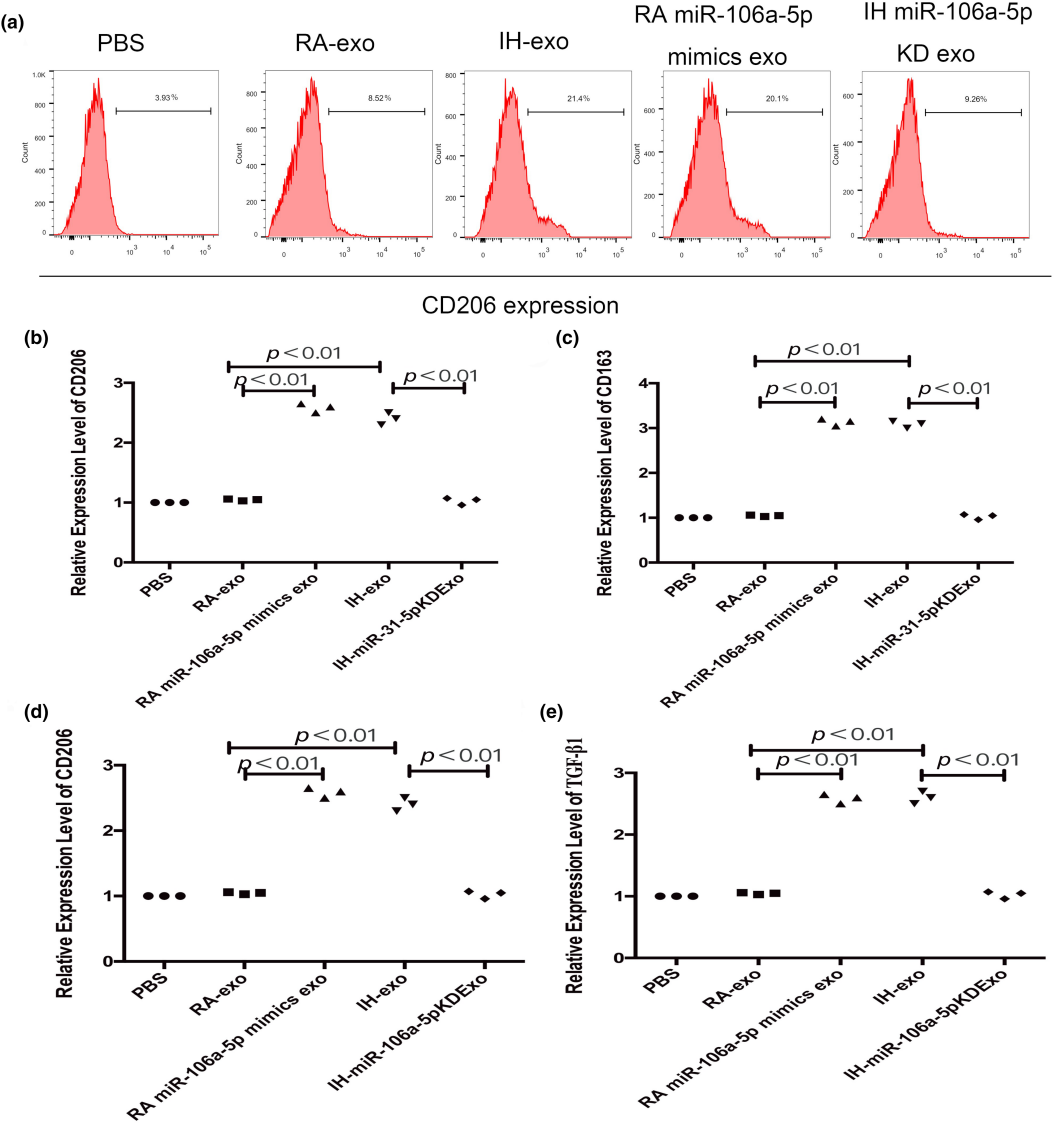
Macrophages picked up from tumor tissue present as a M2‐like phenotype prominently. (a) The proportion of CD206+ cells was detected by flow cytometry in macrophages harvested from tumor tissues in mice. (b–e) Expression levels of TGF‐β1, CD206, CD163 and Arginase‐1 mRNA were detected by RT‐qPCR in macrophages of tumor tissues in mice. KD, knockdown. *n* = 3.

The flow cytometric assay displayed a higher ratio of CD206+ cells in macrophages derived from IH exosomes or RA miR‐106a‐5p mimics exosomes group than RA exosomes group, which was reversed by inhibition of miR‐106a‐5p in IH exosomes (Figure 11a). RT‐qPCR showed significantly higher levels of TGF‐β1, CD206, CD163 and Arginase‐1 mRNA, which indicates an M2‐like immunophenotype, in macrophages selected from IH exosomes or RA miR‐106a‐5p mimics exosomes group than RA exosomes group, which was abolished by inhibition of miR‐106a‐5p in IH exosomes (Figure 11b–e).

### 3.8 AHI is correlated with the expression of miR‐106a‐5p in serum exosomes and poor prognosis in patients of OSA complicated with NSCLC

**FIGURE 12:**
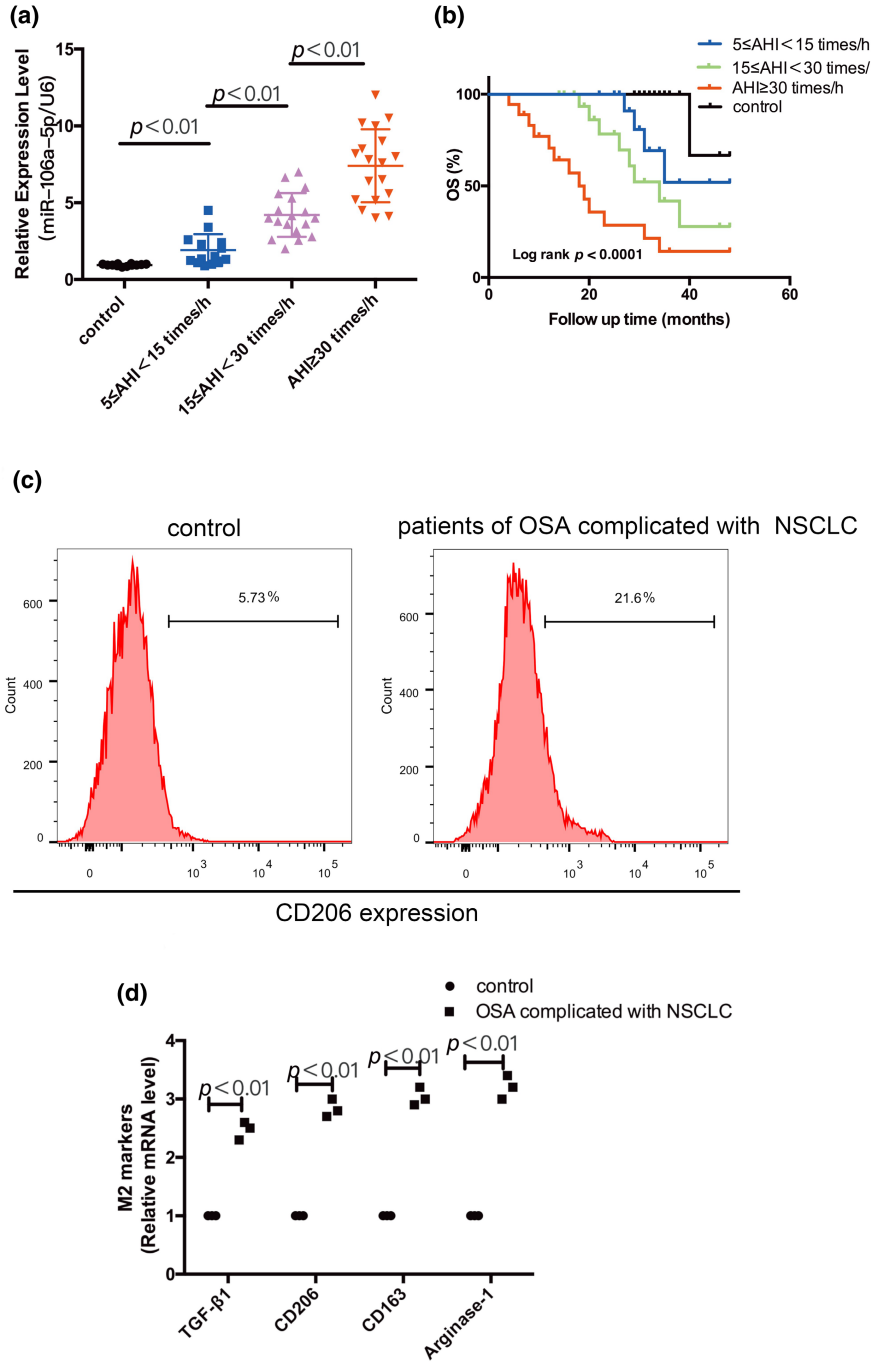
AHI affected the expression of miR‐106a‐5p in serum exosomes and the prognosis in patients of OSA complicated with stage II/III NSCLC. (a) Expression of miR‐106a‐5p in serum exosomes of patients with different severity of apnea. (b) A Log‐rank analysis of OS for patients of OSA complicated with stage II/III NSCLC with the AHI. (c) The proportion of CD206+ cells was detected by flow cytometry. (d) Expression levels of TGF‐β1, CD206, CD163 and Arginase‐1 mRNA were detected by RT‐qPCR in macrophages of peripheral origin. *n* = 3.

To analyze the correlation between miR‐106a‐5p expression and clinicopathological parameters in patients with OSA complicated with stage II/III NSCLC. All of the included patients were dichotomized into two groups (high expression group:>median score; low expression group:≤median score) based on the median score of miR‐106a‐5p expression. As shown in Table 1, there was no difference between patients and healthy volunteers, in age, gender, and pathological type, circulating exosomal miR‐106a‐5p levels were closely associated with AHI. To further study the correlation between exosomal miR‐106a‐5p and the AHI, serum exosomal miR‐106a‐5p expression was measured by PCR, and the expression of serum exosomal miR‐106a‐5p is positively correlated with AHI (Figure 12a). Follow‐up of patients with OSA and stage II/III NSCLC revealed that more severe AHI classification significantly associated with worse OS (overall survival) (*p* < 0.0001) (Figure 12b). Next, we wondered whether the characterized phenotype of peripheral monocytes is different in patients of OSA complicated with NSCLC and healthy controls, and the ratio of CD206+ cells was shown to be strikingly higher in macrophages collected from the peripheral blood of patients of OSA complicated with NSCLC (Figure 12c). RT‐qPCR results also showed higher mRNA levels of TGF‐β1, CD206, CD163, and Arginase‐1 in peripheral blood‐derived macrophages selected from patients of OSA and NSCLC than healthy controls (Figure 12d).

## 4 DISCUSSION

OSA is closely related to the morbidity and mortality of cancer (Gozal et al., 2016; Kendzerska et al., 2014; Martinez‐Garcia et al., 2014), IH, a characteristic of OSA, also contributes to tumor migration and invasion in animals (Almendros et al., 2012, 2013). However, the mechanism by which IH promotes tumor development is still unclear.

Tumor‐associated macrophages (TAMs) are the most common immune‐related tumor microenvironment (TME) stromal cells (Chanmee et al., 2014; Noy & Pollard, 2014). Two subtypes of TAMs, M1 and M2, exhibited anti‐tumor and pro‐tumor properties, respectively (Mantovani & Locati, 2013). M2 macrophages promote tissue repair and remodeling, angiogenesis, and immunosuppression, and thus can promote tumor progression (Biswas et al., 2013; Guo et al., 2013). Almendros et al. found that IH promotes the growth of TC1 tumors in vivo through leading a decrease in the macrophage M1/M2 ratio (Almendros et al., 2014).

Exosomes are lipid bilayer vesicles containing amino acids, proteins, and miRNAs that can be secreted by various cells (Raposo & Stoorvogel, 2013). Many studies have shown that exosomes are associated with tumor progression (Wu et al., 2018, 2019; Zhang, Sai, et al., 2019). Previous studies have shown that both circulating exosomes in OSA could promote the proliferation, migration and invasion of TC1 lung adenocarcinoma cells (Almendros et al., 2016). However, the effect of tumor cell‐derived exosomes on tumors under OSA has not been studied.

MicroRNAs could inhibit the transcription of mRNA by binding to the 3′‐UTR of the target mRNA, ultimately regulating the expression of oncogenic or tumor‐suppressor genes, thus promoting tumor development (Mao & Wang, 2015; Zhou et al., 2017). MicroRNAs promote tumor migration and invasion by interacting with stromal cells in the TME, such as macrophages (Zang et al., 2019). Accumulating evidence has revealed that tumor‐derived exosomes frequently transfer miRNAs to recipient cells to induce repression of the target genes (Wang et al., 2020; Zhang et al., 2015). However, fewer studies have focused on the transfer of NSCLC‐derived exosomal miRNAs to macrophages and their subsequent role in the malignancy of recipient cells when concomitant NSCLC and OSA.

The present study, which utilized an IH model that mimicked OSA‐related changes, demonstrated that IH promotes M2 polarization of macrophages through exosomal miR‐106a‐5p and then increases the proliferation, migration and invasion abilities of NSCLC in vitro and in vivo.

STAT3 is a key regulator of multiple cellular processes, such as proliferation, immune function, and angiogenesis. Normal STAT3 signaling is tightly controlled in standard cellular responses, but constitutive STAT3 activation often occurs in a variety of human cancers, especially lung cancer (Harada et al., 2014; Iriki et al., 2017). STAT3 also plays an important role in immune response by regulating immune checkpoint proteins and tumor environmental cytokines (Tong et al., 2020). Activation of STAT3 is involved in the polarization of M2 macrophages (Degboe et al., 2019; Ma et al., 2019; Wen et al., 2018), and promotes tumor development (Degboe et al., 2019; Ye et al., 2019; Zhang, Li, et al., 2019), and the research of the effect of STAT3 on macrophage polarization has obtained attention in recent years (Xiong et al., 2012). Moreover, STAT3 plays a crucial role in regulating the EMT process in several cancer types. In addition, as a tumor‐suppressor gene, PTEN can inhibit tumor invasion and metastasis by enhancing the adhesion ability and inhibiting the degradation of extracellular matrix and tumor angiogenesis (Shi et al., 2019). We confirmed that miR‐106a‐5p could directly bind to PTEN. Using RNA interference and overexpressing cell lines, we illustrated that IH‐induced polarization of M2 macrophages through up‐regulation of STAT3 signaling pathway or down‐regulation of PTEN. These changes were attenuated by inhibition of STAT3 or overexpression of PTEN.

In addition, we demonstrated that OSA increased the expression of exosomal miR‐106a‐5p and promoted the polarization of M2 macrophages in patients. The patients of OSA and NSCLC had significantly higher levels of circulating exosomal miR‐106a‐5p and the level of circulating exosomal miR‐106a‐5p was correlated positively with AHI. And the phenotype of monocytes in peripheral blood was more inclined to be re‐polarized to an M2 subtype in patients of NSCLC and OSA than healthy controls. And follow‐up of patients with OSA and stage II/III NSCLC showed that more severe AHI grade was associated with shorter overall survival.

Both lung adenocarcinoma and lung squamous cell lines were employed to verify the current findings. Also, the clinical samples were also employed to demonstrate the potential and importance of exosomal miR‐106a‐5p blockade in the treatment of patients with OSA and concurrent NSCLC.

Our results demonstrated that IH exposure plays an important role in promoting NSCLC progression, partially through exosomal miR‐106a‐5p and increasing TAMs population. Hence, targeting exosomal miR‐106a‐5p might be a good strategy to treat NSCLC patients with OSA.

## AUTHOR CONTRIBUTIONS

Jie Ren conceived the study and contributed to data analysis; Yongjie Huang and Zhuan Jin performed the experiments and contributed to drafting the manuscript; all authors reviewed and approved the final version of the manuscript.

## FUNDING INFORMATION

This work was supported by the National Natural Science Foundation of China (grant 82100106).

## CONFLICT OF INTEREST STATEMENT

The authors have no conflicts of interest to declare.

## ETHICS APPROVAL AND CONSENT TO PARTICIPATE

All animal experiments were approved by the Institutional Animal Care and Use Committee (IACUC). Human experiments were approved by the Institutional Review Board of The First Affiliated Hospital of Zhengzhou University.

## Data Availability

The analyzed datasets generated during the study are available from the corresponding author on reasonable request.
